# Crop‐to‐wild gene flow and spatial genetic structure in the closest wild relatives of the cultivated apple

**DOI:** 10.1111/eva.12059

**Published:** 2013-06-28

**Authors:** Amandine Cornille, Pierre Gladieux, Tatiana Giraud

**Affiliations:** ^1^ CNRS Laboratoire Ecologie Systématique et Evolution – UMR8079 Orsay France; ^2^ University Paris Sud Orsay France; ^3^ Department of Plant and Microbial Biology University of California Berkeley CA 94720‐3102 USA

**Keywords:** apple scab, conservation, costructure, hybridization, pathogen, tree, *Venturia inaequalis*

## Abstract

Crop‐to‐wild gene flow have important evolutionary and ecological consequences and require careful consideration in conservation programs for wild genetic resources of potential use in breeding programs and in assessments of the risk of transgene escape into natural ecosystems. Using 26 microsatellites and a set of 1181 trees, we investigated the extent of introgression from the cultivated apple, *Malus domestica*, to its three closest wild relatives, *M. sylvestris* in Europe, *M. orientalis* in the Caucasus, and *M. sieversii* in Central Asia. We found footprints of introgression from *M. domestica* to *M. orientalis* (3.2% of hybrids), *M. sieversii* (14.8%), and *M. sylvestris* (36.7%). *Malus sieversii* and *M. orientalis* presented weak, but significant genetic structures across their geographic range. *Malus orientalis* displayed genetic differentiation with three differentiated populations in Turkey, Armenia, and Russia. *Malus sieversii* consisted of a main population spread over Central Asia and a smaller population in the Tian Shan Mountains. The low *Sp* values suggest high dispersal capacities for the wild apple relatives. High potential for crop‐to‐wild gene flow in apples needs to be considered in the implementation of *in situ* and *ex situ* actions for the conservation of wild apple genetic resources potentially useful to plant breeding.

## Introduction

The anthropogenization of landscapes and ecosystems, with landscape fragmentation and the introduction of crops over extended areas, has greatly increased the likelihood of contact between domesticated and related wild taxa that were previously isolated either geographically or ecologically (Kareiva et al. 2007). In the last 20 years, an increasing number of studies have documented introgression from crops into their wild or weedy relatives (Ellstrand et al. 1999; Arnaud et al. 2003; Ellstrand 2003). Crop‐to‐wild gene flow thus appears to be more frequent than previously thought on the basis of the assumption that domesticated traits probably reduce fitness in natural conditions (Ellstrand 2003). Thirteen major food crops have been shown to hybridize with their wild relatives (Ellstrand et al. 1999). These hybridization events are facilitated by the frequent lack of a strong intrinsic reproductive barrier between domesticated crops and their wild relatives (Ellstrand et al. 1999; Gepts and Papa 2003).

Crop‐to‐wild introgression may greatly affect the evolution and ecology of wild relatives of domesticated plants (Ellstrand 1992), making this subject area of investigation a top priority for conservation. The most direct negative consequences of crop‐to‐wild gene flow include a loss of wild‐population integrity—already jeopardized by a loss of within‐species genetic diversity due to fragmentation (Sagnard et al. 2011)—resulting in a potential loss of gene pools important for ecosystem function (Ellstrand 2005). Indirectly, detailed investigations of gene flow between crops and wild relatives are also required for the development of conservation plans for wild crop relatives as genetic resources for breeding purposes, wild gene pools being potentially enriched in alleles, genes, or gene complexes that can be favorable to agriculture (Feuillet et al. 2008). Crop‐to‐wild gene flow investigation is also germane to the debate over the possible effect of transgene flow into natural ecosystems (Ellstrand 2003; Papa 2005). However, hybridization and introgression rates vary considerably between populations and species (Ellstrand 2003), hindering the development of general conservation programs. There is therefore an urgent need to quantify crop‐to‐wild relative gene flow for the various domesticated species and their wild relatives.

Dispersal capacity and the extent of gene flow between wild relative populations strongly affect the spread and ultimate distribution of domesticated alleles or transgenes in the landscape. It is thus important to evaluate the dispersal capacities of wild crop relatives exposed to seed and pollen flow from domesticated taxa, for the evaluation of potential crop‐to‐wild gene flow (Krutovsky et al. 2012). Dispersal capacities can be estimated indirectly by analyzing intraspecies spatial genetic structure (Vekemans and Hardy 2004). In programs aiming at conserving wild genetic resources, the characterization of spatial genetic structure and of levels of introgression between species is essential for the designation of the best populations to be targeted for conservation *in situ* (e.g., conservation of several genetically differentiated populations) or *ex situ* (e.g., establishment of orchards or seed‐based core collections from pure wild individuals, maximizing genetic diversity). In breeding programs, genetically differentiated populations are often used to improve the local adaptation of cultivars or to enhance resistance to pathogens of cultivars, by selecting for sources of disease resistance from wild populations (Lenne and Wood 1991; Feuillet et al. 2008).

Trees generally have exceptionally high long‐distance dispersal capacities (Kremer et al. 2012) and are therefore important models for studies of crop‐to‐wild gene flow. Gene flow through long‐distance dispersal may increase the likelihood of hybridization between wild and crop populations, thereby increasing the risk of introgression of domesticated alleles or potential transgenes over a large scale. Interest in gene flow from cultivated trees to their wild relatives has increased in the last decade (e.g., Duputié et al. 2007; Delplancke et al. 2011; Miller and Gross 2011) with trees and shrubs accounting for 16% of the 48 plant species for which substantial evidence of crop‐to‐wild gene flow was obtained over this period (Ellstrand 2003).

The cultivated apple (*Malus domestica*) is one of the most widely grown fruit crops of temperate regions, with an annual worldwide production of about 70 million tons (http://faostat.fao.org/). Apple trees are self‐incompatible, favoring intra‐ and interspecific gene flow. The cultivated apple was domesticated in Central Asia from *Malus sieversii* (Velasco et al. 2010; Cornille et al. 2012) and was brought to Europe through human migrations about 3000 years ago (Juniper and Mabberley 2006). The migration of domesticated apples westwards from Asia may have involved contact and hybridization with local wild *Malus* relatives growing along the Silk Route. Wild‐to‐crop gene flow has been previously evidenced during the evolutionary history of the cultivated apple, with a major contribution of the European crabapple, *M. sylvestris*, to the genetic makeup of modern domesticated apples, and a possible contribution of the Caucasian apple *M. orientalis* to the genome of some Mediterranean cultivars (Cornille et al. 2012). These three wild species are small insect‐pollinated trees of the Rosaceae family. *Malus sylvestris* and *M. orientalis* grow in low‐density populations in natural habitats, whereas *M. sieversii* forms high‐density populations in the Tian Shan Mountains (Jackson and Weng 1999). The three wild relatives are mostly pollinated by bees and flies (Syrphidae). Diverse wild animals, including mammals and large birds, feed on the apple fruit, but their respective efficiencies as seed‐dispersal vectors are unknown (Juniper and Mabberley 2006; Larsen et al. 2006). Hybrids cannot be differentiated from cultivated apples or pure wild individuals on the basis of morphological characteristics alone, molecular tools are therefore required to investigate hybridization. Molecular studies investigating the extent of hybridization between *M. domestica* and *M. sylvestris* gene pools have identified a few wild *M. sylvestris* trees displaying introgression from *M. domestica* in populations from Denmark and Belgium (Coart et al. 2006; Larsen et al. 2006; Larsen and Kjær 2009). However, we still know little about crop‐to‐wild gene flow, spatial genetic structure, and dispersal capacities across the full geographic ranges of the wild relatives that have contributed to the cultivated apple genome.

We used 26 microsatellite markers and a comprehensive set of 1181 samples of three wild apple species collected in Europe, the Caucasus, and Central Asia and a set of pure *M. domestica* reference previously identified (Cornille et al. 2012) to investigate the extent of crop‐to‐wild gene flow from the cultivated apple to its wild relatives *M. sieversii*,* M. orientalis* and *M. sylvestris* (Cornille et al. 2012). We also investigated the spatial genetic structure and dispersal capacities of the three wild relatives of the cultivated apple. The spatial genetic structure and dispersal capacity of the European wild apple, *M. sylvestris*, have recently been investigated (Cornille et al. 2013) and are therefore not presented here.

## Materials and methods

### Sampling, DNA extraction, and microsatellite genotyping

Leaf material was collected from (i) *M. sylvestris* (*N *=* *796) at 56 sites across Europe; (ii) *M. orientalis* (*N *=* *217) at 27 sites across the Caucasus: mainly in Armenia (25 sites) and two additional sites at the extreme geographical range of *M. orientalis*, in Russia (1 site) and Turkey (1 site); (iii) *M. sieversii* at 28 sites across Central Asia (Kazakhstan, China, Kyrgyzstan, Tajikistan, and Uzbekistan) (*N *=* *168). Details of the sampling sites are provided in Table S1. We chose 40 *M. domestica* individuals previously identified as displaying no introgression from *M. sylvestris*,* M. sieversii,* or *M. orientalis*, that is, with membership coefficients exceeding 0.9 for the *M. domestica* gene pool in a STRUCTURE analysis (Cornille et al. 2012). These 40 individuals were used as the reference ‘pure’ *M. domestica* gene pool, for the identification of hybrids between wild species and the domesticated apple.

DNA was extracted using the Nucleo Spin^®^ plant DNA extraction kit II (Macherey & Nagel, Düren, Germany). Multiplex microsatellite PCR amplifications were performed using a Multiplex PCR Kit (Qiagen, Inc., Courtaboeuf, France). We used 26 microsatellites distributed across the 17 chromosomes (one to three microsatellites per chromosome) using 10 different multiplex reactions, as previously described (Patocchi et al. 2009; Cornille et al. 2012). We retained only multilocus genotypes with less than 20% missing data.

### Bayesian inference of introgression and population structure

We used the individual‐based Bayesian clustering methods implemented in STRUCTURE 2.3.3 (Pritchard et al. 2000) to estimate introgression from *M. domestica* into the wild species. STRUCTURE makes use of Markov Chain Monte Carlo (MCMC) simulations to infer the proportion of ancestry of genotypes from *K* distinct clusters. The underlying algorithms attempt to minimize deviations from Hardy–Weinberg and linkage disequilibria. Preliminary Bayesian analyses with STRUCTURE showed that the most relevant numbers of populations (*K*_*w*_) for the wild species were *K*_*w*_ = 3 for *M. sylvestris* (Cornille et al. 2013); *K*_*w*_ = 2 for *M. sieversii,* and *K*_*w*_ = 3 for *M. orientalis* (data not shown). We then used STRUCTURE on three data sets, each including the 40 reference *M. domestica* genotypes and one wild species, setting the number of clusters (*K*) to values corresponding to the number of clusters (*K*_*w*_) determined in preliminary analyses on the focal wild species, plus one for *M. domestica*, that is, *K* = *K*_*w*_ + 1, with *K *=* *3 for *M. sieversii* and *K *=* *4 for both *M. orientalis* and *M. sylvestris*. We considered that individuals assigned to a wild gene pool with a membership coefficient <0.9 were introgressed by *M. domestica*. The admixture proportion of a wild genotype assigned to the *M. domestica* gene pool is denoted *P*_*domestica*_.

For the analysis of within‐species population structure, we excluded hybrids previously detected with STRUCTURE analyses, and used the individual‐based spatially explicit Bayesian clustering method implemented in TESS 2.3.1 (Chen et al. 2007). These analyses were performed for *M. sieversii* and *M. orientalis* only, as the population structure of *M. sylvestris* has already been investigated (Cornille et al. 2013). TESS also makes use of MCMC simulations to infer the proportion of ancestry of genotypes from *K* distinct clusters, with the underlying algorithms attempting to minimize deviations from Hardy–Weinberg and linkage disequilibria. TESS also incorporates a spatial component into the clustering procedure, such that genotypes from areas located closer together geographically are considered more likely to belong to the same cluster. In TESS analyses, we used the conditional autoregressive (CAR) Gaussian model of admixture with linear trend surface, setting the spatial interaction parameter (*ρ*) to 0.6. These parameters (*ρ* and trend) affect the weight given to spatial distance when clustering genotypes. Ten independent analyses were carried out for each number of clusters *K* (2 ≤ *K *≤* *6), using 500 000 MCMC iterations after a burn‐in of 50 000 steps. Outputs were processed using CLUMPP v1.1.2 (Jakobsson and Rosenberg 2007) to identify distinct modes (i.e., clustering solutions) in the replicated runs of each *K*.

### Genetic variation within *Malus sieversii* and *Malus orientalis*

For the analysis of within‐species genetic variation, we excluded hybrids previously identified as introgressed by *M. domestica* (i.e., individuals assigned to a wild gene pool with a membership coefficient <0.9).

We used ARLEQUIN (Excoffier and Lischer 2010) to check the suitability of the markers for population genetic analyses in each species. None of the 26 microsatellite markers significantly deviated from a neutral equilibrium model, as shown by the nonsignificant *P*‐values obtained in Ewens–Watterson tests, and no pair of markers was in significant linkage disequilibrium (Raymond and Rousset 1995; Rousset 2008). The markers were therefore considered to be unlinked and to be subject to quasi‐neutral evolution, in each species.

We tested for the occurrence of null alleles using MICRO‐CHECKER 2.2.3 (Van Oosterhout et al. 2004). Allelic richness was calculated using ADZE software (Szpiech et al. 2008) for sites (i.e., geographic locations), using sample sizes of *N *=* *14 (seven individuals × two chromosomes) for *M. orientalis* and *N *=* *6 (three individuals x two chromosomes) for *M. sieversii*, corresponding to the smallest number of observations for sites. Heterozygosity, Weir and Cockerham *F*‐statistics, and Hardy–Weinberg genotypic linkage equilibrium were assessed using GENEPOP 4.0 (Raymond and Rousset 1995; Rousset 2008). Only sampling sites with at least six successfully genotyped specimens were included in site‐specific calculations (seven sites for *M. sieversii* and 14 for *M. orientalis*).

We checked for isolation‐by‐distance (IBD) patterns, as previously described (Loiselle et al. 1995). A Mantel test with 10 000 random permutations was performed between the individual coefficient of relatedness *F*_ij_ and the matrix of the natural logarithm of geographic distance. These analyses were performed using SPAGeDI 1.3 (Hardy and Vekemans 2002), separately for the main populations (i.e., clusters identified by TESS analyses), in *M. sieversii* and *M. orientalis* (see Results). Because the inclusion of genotypes with high levels of *M. domestica* ancestry may strongly bias estimated patterns of relatedness, only individuals having membership coefficients above 0.55 in the cluster under consideration were included in these analyses.

## Results

### Estimation of introgression from the cultivated apple to the wild species

**Figure 1:**
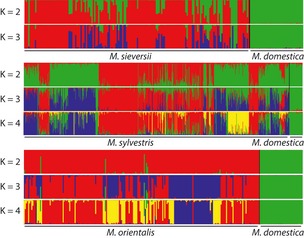
Coefficients of membership in various gene pools inferred with the STRUCTURE program, based on data sets including 40 *Malus domestica* reference samples (green, *N *=* *40) and one of the three wild *Malus* species in each case. The *x*‐axis is not shown in scale. Hybrids were detected by running STRUCTURE from *K *=* *2 to *K *=* *3 for *Malus sieversii* and up to *K *=* *4 for *Malus sylvestris* and *Malus orientalis*.

STRUCTURE analyses consistently detected hybrid genotypes in wild species (Fig. 1). The assignments obtained at lower *K* values confirmed that running STRUCTURE at *K* values below the most relevant *K* value can create spurious assignments (Kalinowski 2011) (Fig. 1). For instance, some of the *M. sieversii* individuals partly assigned to the *M. domestica* gene pool at *K *=* *2 actually formed a distinct pure *M. sieversii* population at *K *=* *3, without footprints of admixture with the *M. domestica* gene pool. Not taking into account the population structure of the wild species can thus lead to the detection of spurious footprints of introgression (Kalinowski 2011). Setting *K* at higher values revealed no further substructure within species, indicating that the admixture detected at *K*_*w *_+ 1 was not an artifact.

**Figure 2:**
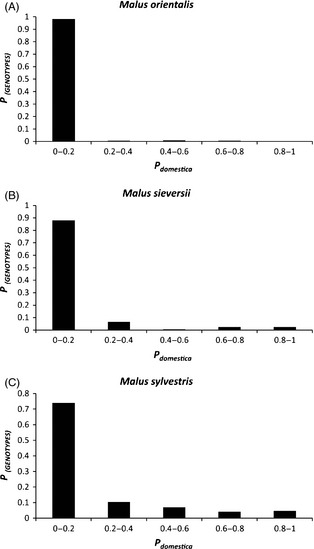
Distribution of the admixture proportion of wild genotypes to the *Malus domestica* gene pool (*P*_*domestica*_) inferred by STRUCTURE for (A) *Malus orientalis* at *K *=* *4, (B) for *Malus sieversii* at *K *=* *3 (C) for *Malus sylvestris* at *K *=* *4. *P*_*domestica*:_ admixture proportion of a wild genotype assigned to the *M. domestica* gene pool, average across sites for each class, *P*_(GENOTYPES)_: proportion of genotypes.

Each wild species included genotypes showing signs of *M. domestica* ancestry (i.e., individuals with membership coefficients <0.9 in the wild‐species gene pool) (Fig. 2). However, the proportion of hybrids differed considerably between the wild species: seven hybrid genotypes were identified in *M. orientalis* (3.2% of the sample), 25 hybrids were identified in *M. sieversii* (14.8% of the sample), and *M. sylvestris* included many hybrids, with 292 admixed individuals (36.7% of the sample). Misidentified individuals (i.e., pure *M. domestica* genotypes) were detected within *M. sylvestris* (*N *=* *37) and *M. sieversii* (*N *=* *4). The distribution of *P*_*domestica*_ *,* averaged across sites for each *P*_*domestica*_ class (i.e., 0–0.2; 0.2–0.4; 0.4–0.6; 0.6–0.8; 0.8–1) and for each wild species is presented in Fig. 2. The distribution of admixture proportions varied among wild species (χ² = 74.6, *P *<* *0.01). All *M. orientalis* trees displaying introgression from *M. domestica* had low proportions of admixture, *M. sieversii* included some individuals with higher admixture proportions, whereas *M. sylvestris* presented the whole range of admixture values (Fig. 2). *Malus sylvestris* presented a large number of intermediate hybrid genotypes, with trees displaying introgression from *M. domestica* (0.1 < *P*_*domestica *_< 0.45), and even included trees that appeared to be hybrids backcrossed with *M. domestica* or feral *M. domestica* displaying introgression from *M. sylvestris* (0.55 < *P*_*domestica *_< 0.9) across its distribution range.

### Population subdivision and IBD in *Malus sieversii* and *Malus orientalis*

Summary statistics for population structure and diversity are shown for *M. sieversii* and *M. orientalis* in Table 1. After the removal of hybrids (i.e., genotypes assigned to a wild gene pool with a membership coefficient <0.9), respectively eight sites and 14 sites had at least six samples available in *M. sieversii* and *M. orientalis* (average sample sizes: 10.9 ± 6.1 and 12.9 ± 3.5 genotypes respectively). On average, across sites and markers, allelic richness (*A*_R_) and gene diversity (*H*_E_) were *A*_R_ = 3.5 ± 0.3 [3.1–3.9] and *H*_E_ = 0.75 ± 0.03 [0.69–0.76] in *M. sieversii*, and *A*_R_ = 6.2 ± 0.4 [5.4–7.1] and *H*_E_ = 0.81 ± 0.02 [0.69–0.76] in *M. orientalis*. *F*_IS_ values were low, with a mean of 0.02 ± 0.04 per site and marker for *M. sieversii* and 0.04 ± 0.06 for *M. orientalis*. The species‐wide heterozygote deficit was highly significant (*P *<* *0.001), but low (*F*_IS_ = 0.05 for *M. sieversii*,* F*_IS_ = 0.06 for *M. orientalis*). Between‐site differences in allelic frequencies, estimated from the mean *F*_ST_ across loci, were low (*F*_ST_ = 0.02; −0.008 to 0.06 for *M. sieversii*,* F*_ST_ = 0.02; −0.02 to 0.07 for *M. orientalis*), but significant for respectively 15 and 58 pairs of sites for *M. sieversii* and *M. orientalis* (*P *<* *0.05, Table S2).

**Table 1:** Genetic variation within *Malus sieversii* and *Malus orientalis*

Species	Site	*N*	*H*_O_	*H*_E_	*F*_IS_	*A*_R_
*M. sieversii* a	Ch Xinj	21	0.71	0.75	0.05^b^	3.5
	Kaz 3	8	0.76	0.74	−0.03	3.5
	Kaz Aksu	7	0.72	0.72	0.007	3.9
	Kaz Kuz	13	0.69	0.72	0.05^b^	3.1
	Kaz djun res	19	0.7	0.80	0.05	3.5
	Kaz tauturgen	7	0.75	0.78	0.04^a^	3.2
	Kaz unid 2	6	0.71	0.77	0.07	3.9
	Overall	101	0.73	0.77	0.05^b^	
*M. orientalis* b	ARA	10	0.82	0.81	−0.02	6.1
	Djermuk 1	10	0.71	0.82	0.14^b^	5.7
	Djermuk 2	9	0.74	0.77	0.03	5.4
	Hermon 1	14	0.81	0.80	−0.02	6.5
	Hermon 2	12	0.78	0.81	0.04^b^	6.2
	Hermon 3	12	0.84	0.80	−0.06	6.1
	Jermouck	13	0.77	0.84	0.08^b^	7.1
	Khosrov Reserve 1	10	0.74	0.85	0.13^b^	6.5
	Khosrov Reserve 5	8	0.81	0.84	0.03	6.5
	Khosrov Reserve 6	15	0.80	0.82	0.03	6.3
	Shikahogh 1	16	0.76	0.83	0.08^b^	6
	Shikahogh 3	16	0.79	0.81	0.03	6
	Vorotanpass 1	15	0.80	0.83	0.04^b^	6.3
	Vorotanpass 2	21	0.77	0.78	0.02	5.9
	Overall	211	0.79	0.83	0.06^b^	

*N*, sample size of each cluster, *H*_O_ and *H*_E_, observed and expected heterozygosities, *F*_IS_, inbreeding coefficient, *A*_R_, mean allelic richness across loci, corrected by the rarefaction method.

a For *Malus sieversii*, five sites (*Ch pop1*,* Kaz Kokbu*,* Kaz Kotyr*,* Uzbe*,* Kaz Almat*) with *N *<* *6 were excluded from these analyses. 0.05 < *P *≤* *0.01.

b For *Malus orientalis,* 14 sites (*KhosrovReserve 2*,* 3*,* 3bis*,* 3ter*,* 4*,* 7*,* 8, 9*,* unidentified*,* Arm unknown*,* Kaz Kokbu*,* Turkey*,* Russia*,* Shikahogh2*) with *N *<* *6 were excluded from the analyses. *P *<* *0.001.

**Figure 3:**
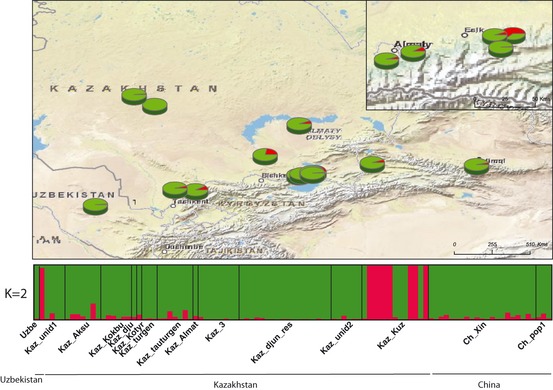
Bayesian clustering results for *Malus sieversii* (*N* = 101) in Central Asia, obtained with TESS at *K* = 2, and associated map of mean membership probabilities per site. Each individual is represented by a vertical bar, partitioned into *K* segments representing the amount of ancestry of its genome corresponding to K clusters. Visualization was improved by sorting genotypes by site.

Spatially explicit clustering analyses with TESS revealed two and three well‐defined clusters for *M. sieversii* and *M. orientalis*, even when setting *K *>* *2 (Fig. S1) and *K *>* *3 (Fig. S2), respectively. We thus concluded that *K *=* *2 and *K *=* *3 were the biologically most relevant numbers of populations, for *M. sieversii* and *M. orientalis,* respectively, as observed in preliminary analyses with full data sets. For *M. sieversii*, clustering patterns at *K *=* *2 revealed a large cluster spreading across Central Asia (green) and a small cluster (red) consisting mostly of individuals from the *Kaz Kuz* population in the Tian Shan Mountains (Fig. 3). The two populations are referred to as the ‘Mountains’ (red at *K *=* *2, *N *=* *9) and ‘Main’ (green at *K *=* *2, *N *=* *92) populations.

**Figure 4:**
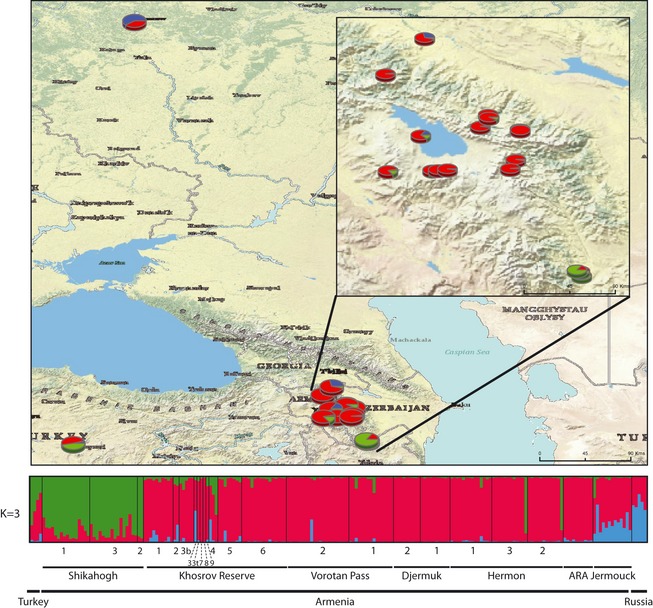
Bayesian clustering results for *Malus orientalis* in the Caucasus (*N* = 217) obtained with TESS at *K* = 3 and an associated map of mean membership probabilities per site. Each individual is represented by a vertical bar, partitioned into *K* segments representing the amount of ancestry of its genome corresponding to *K* clusters. Visualization was improved by sorting the genotypes by site.

For *M. orientalis*, clustering patterns at *K *=* *2 indicated a north/south genetic differentiation between a cluster encompassing the Turkish and Armenian *Shikahogh* populations (green) and another cluster comprising most of the Armenian populations (red) (Fig. S2). At *K *=* *3, a third cluster (blue) appeared, including the Russian population and the Armenian *Jermouck* population (Fig. 4). Six genotypes could not be assigned to any population and were therefore not included in subsequent IBD analyses. The three populations identified in *M. orientalis* are referred to hereafter as the ‘Northern’ (blue, *N *=* *4), the ‘Central’ (red, *N *=* *159), and the ‘Southern’ (green, *N *=* *38) populations.

Within the three populations for which *N *>* *10 (i.e., the ‘Central’ and ‘Southern’ population for *M. orientalis* and the ‘Main’ population for *M. sieversii*), genetic differentiation and geographic distance were significantly correlated, consistent with an IBD model. The *Sp* statistic can be used to quantify spatial structure and is useful for comparisons between populations and/or species. Lower *Sp* values are associated with greater dispersal capacities and/or effective population sizes. *Sp* values were very low, close to 0, but were significant in the *M. sieversii* population (*Sp* = −0.002, *P *<* *0.05) and in *M. orientalis* populations (‘Central’: *Sp* = 0.003, *P *<* *0.001; ‘Southern’: *Sp* = 0.002*, P < *0.001). These results suggest that the wild *Malus* species have high dispersal capacities and/or a large effective population size.

## Discussion

Wild relatives of the cultivated apple represent a valuable genetic resource for enriching and improving the cultivated apple gene pool, by incorporating alleles or allelic combinations providing greater tolerance to abiotic factors or resistance to pest and diseases. Knowledge of the population structure and dispersal capacities of wild apples and of the degree of hybridization of these wild species with the domesticated apple is essential for the development of sound conservation plans and innovative breeding strategies. Our findings reveal substantial gene flow from *M. domestica* to wild apple relatives from Europe and the Caucasus (*M. sylvestris* and *M. orientalis*) and to the main Asian progenitor of cultivated apple (*M. sieversii*). The results obtained in this study, combined with our previous results for the European crabapple (*M. sylvestris*) (Cornille et al. 2013), indicate that the wild apple species have a weak spatial genetic structure, with large‐scale isolation by distance, suggesting high dispersal capacities. Altogether, our findings demonstrate high potential for gene flow from the cultivated apple to its three wild relatives in the future.

### Evidence for crop‐to‐wild gene flow: introgression from *Malus domestica* to its three main wild relatives

Introgression from the cultivated apple to wild relatives has been investigated in *M. sylvestris,* but at a local geographic scale (Coart et al. 2003, 2006; Larsen et al. 2006). Here, we analyzed crop‐to‐wild gene flow across the entire geographic range of three wild *Malus* species, demonstrating much higher introgression levels. Indeed, our results suggest that substantial crop‐to‐wild gene flow occurs: 37% of *M. sylvestris*, 15% of *M. sieversii,* and 3%, of *M. orientalis* samples were found to be hybrids with the cultivated apple. Thus, large‐scale introgression from *M. domestica* to its wild relatives may occur spontaneously. The lower rates of hybridization observed in previous studies in Belgium and Germany for *M. sylvestris* (4%–14%) (Coart et al. 2003, 2006) and in Denmark (2%) may be due to the lower power for detecting the *M. domestica* gene pool in these studies, link to the use of fewer markers and/or smaller reference samples, or potential differences in landscape features (e.g., distance to *M. domestica* orchards or forest fragmentation). Unfortunately, the available information concerning landscape features for our sample was insufficiently detailed for assessments of the influence of specific factors. We also note that our estimates of crop–wild gene flow might be conservative. Admixed individuals were identified using ‘pure’ domesticated apple samples for the crop samples, and we cannot exclude that those chosen trees actually have a lower probability for having donated alleles to the wild.

Previous studies reporting low rates of hybridization between the cultivated apple and the European wild apple (*M. sylvestris*) had suggested that the low reproductive fitness of interspecific hybrids might account for this finding (Coart et al. 2003). By contrast, our findings of high rates of introgression and backcrossing suggest that hybrids may actually be fit in natural habitats. Gene flow from domestic to wild species has been investigated in various plant and animal models and recurrent gene flow from crop to wild species has often been demonstrated (Ellstrand 2003; Arrigo et al. 2011; Delplancke et al. 2011; Sagnard et al. 2011; De Andrés et al. 2012; Goedbloed et al. 2012; Hübner et al. 2012). The proportion of introgressive hybridization found in *M. sylvestris* was of a similar order of magnitude to that reported in wild relatives of other crops, such as goatgrasses (*Aegilops*, 25% of these wild relatives of wheat are hybrids) (Arrigo et al. 2011), wild grapevine (*Vitis vinifera ssp. sylvestris*, 19%) (De Andrés et al. 2012), and prickly lettuce (*Lactuca serriola*, 7%). In wheat, crop‐to‐wild gene flow levels may differ between species due to differences in mating systems, with *Aegilops* species, which are the most allogamous, showing the highest levels of hybridization (Arrigo et al. 2011). In lettuce (*Lactuca sativa*), an allele from the crop, delaying flowering, might confer a selective advantage on hybrids in natural conditions (Hartman et al. 2012).

The previous studies reporting low rates of hybridization in *M. sylvestris* also put forward isolation‐by‐distance as a barrier to hybridization between *M. domestica* and *M. sylvestris* (Larsen et al. 2006). Other factors, such as absence of overlapping flowering times, have also been proposed as potential barriers to hybridization. We show here that the rates of hybridization between *M. domestica* and wild apple trees are much higher than previously thought, suggesting that hybrids are often viable and that potential barriers to interspecific gene flow are actually quite weak. These results raise the question concerning long‐term evolutionary consequences of these crop‐to‐wild introgressions, potentially threatening the genetic integrity of the wild apple relatives.

### Weak spatial genetic structure in wild contributors and high dispersal capacities

The wild apple species *M. sieversii*,* M. orientalis,* and *M. sylvestris* had a weak spatial genetic structure across a wide geographic range, suggestive of high dispersal capacities. The spatial genetic structure of the European wild has been described elsewhere (Cornille et al. 2013). The spatial genetic structure of *M. sylvestris* suggests an ancient contraction followed by expansion since the last glacial maximum in Europe. Three principal populations have been identified in *M. sylvestris*: a large population spreading through Western Europe and two populations with narrower distributions located in the Carpathian Mountains and the Balkans. We investigated the spatial genetic structure of *M. sieversii* and *M. orientalis* across their geographic ranges, in Central Asia and the Caucasus, respectively. Our findings contrast with previous estimates based on fewer samples and markers in these two wild species, which had suggested a much more pronounced regional structure (Volk et al. 2008; Richards et al. 2009).

*Malus sieversii* displayed weak spatial genetic structure over a large geographic scale, with two distinct populations identified: one with a broad distribution throughout Central Asia and the other restricted to the Tian Shan Mountains in Kazakhstan. The differentiation of the *Kaz Kuz* population is particularly interesting when compared with that of the fungal pathogen *Venturia inaequalis*, which causes apple scab disease on the domesticated apple and its wild relatives*. Venturia inaequalis* displays a similar pattern of genetic differentiation in the *M. sieversii* forests of the eastern mountains of Kazakhstan (Gladieux et al. 2010). This correspondence suggests possible costructuring of the populations of the main apple progenitor *M. sieversii* and its pathogen. The *V. inaequalis* population in the Tian Shan Mountains is thought to be a relic of the ancestral populations infecting *M. sieversii* before the domestication of *M. domestica* (Gladieux et al. 2010).

*Malus orientalis* displayed a weak north–south pattern of spatial genetic structure. Albeit the sampling effort was mainly focused in Armenia with few samples from other parts of the Caucasus, three distinct populations were detected: a large population corresponding to most of the Armenian samples and two more narrowly distributed populations, one in the Southern Caucasus (Turkey and southern Armenia) and the other in more northerly latitudes (in Russia).

Isolation‐by‐distance patterns were detected for both *M. orientalis* and *M. sieversii*, as previously reported for *M. sylvestris* (Cornille et al. 2012). The three wild species, with their different geographic distributions and densities, displayed similarly low values of the *Sp* statistic, consistent with high dispersal capacities and large effective population sizes. These values might even be slight overestimates due to the inclusion in analyses of genotypes having up to 45% ancestry in the *M. domestica* gene pool. *Sp* estimates can be used to compare the levels of gene flow between wild apples and other tree species. The *Sp* values of the three wild apple species (all animal dispersed) were of a similar order of magnitude to those estimated for wind‐dispersed trees, such as *Larix laricina*,* Quercus robur,* and *Fraxinus excelsior* (Vekemans and Hardy 2004), but were lower than those found for animal‐dispersed shrub species (e.g., *Sorbus torminalis, Sp* = 0.02).

Several features may account for these high *Sp* values and weak spatial genetic structure. Apples are allogamous, with self‐incompatibility systems favoring gene flow and low levels of population genetic differentiation, as typically observed in trees (Kremer et al. 2012). However, animal seed‐dispersal syndrome might have been expected to limit gene flow (Vekemans and Hardy 2004). Animal dispersers of apples include honeybees, bumblebees, leaf‐cutter bees, and mason‐bees for pollen transport and large mammals, such as wild cattle, brown bear, and humans for seed transport (Juniper and Mabberley 2006). These mammal dispersers can travel over long distances, potentially accounting for the surprising large apple‐dispersal capacities, similar to those found for wind‐dispersed species.

The combined dispersal capacities of the seed and pollen dispersers may account for the low *Sp* values obtained for apple trees, and for the large effective population sizes, typical of trees, reported elsewhere (Cornille et al. 2013). Low density has also been put forward as an explanation for long‐distance dispersal and weak spatial genetic structure (Vekemans and Hardy 2004). *Malus sieversii*,* M. orientalis,* and *M. sylvestris* present variable density populations throughout their geographic ranges (i.e., high‐density populations in the Tian Shan Mountains for *M. sieversii* and scattered populations for *M. orientalis* and *M. sylvestris*), whereas the three wild species had similar *Sp* values. Density thus does not appear to be an important factor underlying the weak spatial genetic structure observed in the three wild apple relatives.

## Conclusion and perspectives

Our findings provide a good picture of the distribution of genetically differentiated wild apple relative populations, an essential component to guide breeding and conservation management programs of the wild relatives and contributors to the cultivated apple genome, *M. orientalis*,* M. sieversii* and *M. sylvestris* (Zhang et al. 2007; Jacques et al. 2009; Pautasso 2009; Velasco et al. 2010; Cornille et al. 2012). In these three wild species, the various genetically differentiated populations identified may harbor a number of different valuable horticultural traits, including resistance to fire blight (*Erwinia amylovora*), resistance to apple scab (*Venturia inaequalis*), fruit quality, and drought tolerance that may serve as gene source for breeding apple programs (Tanksley and McCouch 1997). The discovery of signs of a costructure between *M. sieversii* and *V. inaequalis*, with a small and specific population in the Tian Shan Mountains for both the host and the pathogen species, is of particular interest in terms of breeding programs aiming to increase resistance to apple scab. Albeit our results suggest high levels of historical gene flow throughout the geographic range of *M. sieversii*, contemporary intra‐ and interpopulation gene flow may have been drastically reduced by population fragmentation. *Malus sieversii* is increasingly threatened by forest destruction, with its populations being restricted to areas that have been rapidly decreasing in size over the last decade (Zhang et al. 2007). The conservation of the populations of this important contributor to the domesticated apple across its geographic distribution is thus timely.

The high level of crop‐to‐wild gene flow detected across geographic distributions of the three wild relatives of the cultivated apple, combined with long‐distance intrawild species gene flow and the increasing globalization, points to the likelihood of further introgression from *M. domestica* to its three wild relatives in the near future. Crop‐to‐wild gene flow can lead to gene swamping (Le Normand 2002) and, thus, to the loss of genetic diversity in wild taxa. Such a decline in diversity has been demonstrated in wild *Fragaria virginiana* (Westman et al. 2004)*,* and it has even been suggested that wild cotton, *Gossypium darwinii* and *Gossypium tomentosum,* disappeared through hybridization with the crop *Gossypium hirsutum* (Ellstrand et al. 1999). There is too little information available to predict the possible evolutionary consequences of the introgression of wild apple relatives by the cultivated gene pool in the long term, thus future investigations are needed. A timely challenge is now to evaluate hybrid fitness and to determine whether certain genomic regions are more permeable to gene flow, and whether introgressed alleles are adaptive.

The problem of crop‐to‐wild gene flow is of particular importance in forest trees, given their high dispersal capacity and the associated high risk of transgene flow (Strauss 2011). The spread of transgenes into the wild gene pool might even be increased if the transgene confers a selective advantage to hybrids in wild habitats. For example, transgene spread is thought to have occurred from transgenic rice (*Oryza sativa*) to red rice (*Oryza sativa f. spontanea*), a weed species (Oard et al. 2000). The first genetically modified apples are probably still a long way from approval for market release. However, scientists are already trying to model the risk and impact of transgene flow in apples (Tyson et al. 2011) and our data should prove useful in this endeavor, providing realistic parameter values.

A question that now warrants further investigation is what are the factors influencing the extent of crop‐to‐wild hybridizations in apple. Environmental factors such as the distance of wild populations to *M. domestica* orchards, as well as ecological or geographic factors, may affect hybridization rates. The identification of the factors influencing introgression into wild species would help conservation programs aimed at maintaining the integrity of wild apple populations, which already face several other human threats, including fragmentation, deforestation, and climate change.

## Data archiving

Data deposited in the Dryad repository: doi:10.5061/dryad.dg899.

## Supporting information


**Table S1.** Description of the *Malus* accessions analysed with their geographical origins and providers.
**Table S2.** Pairwise genetic differentiation (*F*_ST_) among *Malus sieversii* sites (*N *>* *6)
**Table S3.** Pairwise genetic differentiation (*F*_ST_) among *Malus orientalis* sites (*N *>* *6)
**Figure S1.** Bayesian clustering results for *Malus sieversii* in Central Asia (*N *=* *101) using the program TESS from *K *=* *2 to *K *=* *6.
**Figure S2.** Bayesian clustering results for *Malus orientalis* in the Caucasus (*N *=* *217) using the program TESS from *K *=* *2 to *K *=* *6.
**Figure S3.** Maps representing the mean membership proportions for *K* clusters, for samples of *Malus sylvestris* collected from the same site.
