# Supplementary material for: Crop‐to‐wild gene flow and spatial genetic structure in the closest wild relatives of the cultivated apple
Source: Evol Appl. 2013 Jun 28;6(5):737–48. doi: 10.1111/eva.12059 (PMC5779123; doi:10.1111/eva.12059)
Supplement: Supplementary file 1 — Table S1. Description of the Malus accessions analysed with their geographical origins and providers. Table S2. Pairwise genetic differentiation (F ST) among Malus sieversii sites (N > 6) Table S3. Pairwise genetic differentiation (F ST) among Malus orientalis sites (N > 6) Figure S1. Bayesian clustering results for Malus sieversii in Central Asia (N = 101) using the program TESS from K = 2 to K = 6. Figure S2. Bayesian clustering results for Malus orientalis in the Caucasus (N = 217) using the program TESS from K = 2 to K = 6. Figure S3. Maps representing the mean membership proportions for K clusters, for samples of Malus sylvestris collected from the same site. [file EVA-6-737-s001.doc]

**Supporting Information**

**Table S1**. Description of the *Malus* accessions analysed with their geographical origins and providers.

**Table S2**. Pairwise genetic differentiation (*F_ST_*) among *Malus sieversii* sites (*N*>6)

**Table S3**. Pairwise genetic differentiation (*F_ST_*) among *Malus orientalis* sites (*N*>6)

Figure S1. Bayesian clustering results for *Malus sieversii* in Central Asia (*N*=101) using the program TESS from *K*=2 to *K*=6.

Figure S2. Bayesian clustering results for *Malus orientalis* in the Caucasus (*N*=217) using the program TESS from *K*=2 to *K*=6.

**Figure S3**. Maps representing the mean membership proportions for *K* clusters, for samples of *Malus sylvestris* collected from the same site.

Table S1. Description of the *Malus* accessions analysed with their geographical origins and providers.

| Species and locations | Nb | Providers |
| --- | --- | --- |
| *Malus sylvestris* | 381 |  |
| Austria | 40 | Thomas Kirisits, Bernhard Kirisits and Heino Konrad |
| Belgium | 9 | Isabel Roldán-Ruiz, CRA-W^1^, ILVO^2^ |
| Bosnia-Herzegovina | 73 | Dalibor Ballian |
| Bulgaria | 1 | Petya Gercheva, Argir Zhivondov, Valentina Bojkova and Anna Matova |
| Denmark | 100 | Anders Larsen |
| France | 45 | INRA^3^, USDA-ARS^4^, Aurélien Cabaret, Nicolas Feau, Jean-Pierre Rioult, Pascal Heitzler |
| Germany | 30 | Jorg Kleinschmit and Wilfried Steiner |
| UK, Scotland | 9 | Stephens Cavers |
| Hungary | 27 | Laszlo Nyari |
| Italy | 5 | Alberto Dominicci and Emanuela Fabrizi, François Salomone, Stephano Porta |
| Norway | 21 | Per Avid |
| Poland | 8 | Jan Kowalczyk and Dzmitry Kahan |
| Romania | 9 | Lucian A. Curtus |
| Spain | 0 | Carlos Ferrera and Francisco Donaire |
| Ukraine | 4 | Roman Volansyanchuk |
| *Malus sieversii* | 168 |  |
| Kazakhstan | 114 | Bruno Le Cam, François Laurens, PG, Emmanuelle Jousselin, Marie-Anne Félix, Catherine Peix and Aymar Dzhangaliev |
|  | 28 | USDA-ARS^4^ |
| China | 26 | Bruno Le Cam, PG, Xiu-Guo Zhang |
| Kirghizstan | 5 | Evelyne Heyer |
| Tajikistan | 1 | USDA-ARS^4^ |
| Uzbekistan | 1 | USDA-ARS^4^ |
| *Malus orientalis* | 217 |  |
| Armenia | 205 | PG, Joanne Clavel, Anush Nersesyan, Ivan Gabrielyan, Ara Hovhannisyan, Karen Manvelyan and Eleonora Gabrielian |
| Russia | 5 | USDA-ARS^4^ |
| Turkey | 5 | USDA-ARS^4^ |
| Unknown | 2 | USDA-ARS^4^ |
| *Malus domestica* | 40 | INRA^3^, CRA-W^1^, USDA-ARS^4^, Dominique Beauvais ^5^ and Jean Pierre Roullaud^7^ |
| Number of trees sampled |  |  |
| ^1^ CRA - W | Centre Wallons de Recherches Agronomiques, Belgium | |
| ^2^ ILVO - PLANT | Plant -Growth and Development, Melle, Belgium | |
| ^3^ INRA | Institut de Recherche en Horticulture et Semences, Angers, France | |
| ^4^ USDA - ARS | Plant Genetic Resources Unit, Geneva (NY) | |
| ^5^ Abbaye de Beauport | Conservatory Orchards of ancient apple varieties, Paimpol, France. | |
| ^6^ EMR | East Malling Research, Kent, UK | |
| ^7^Verger Conservatoire d’Arzano | Conservatory Orchards of ancient apple varieties, Brittany, France. | |

Table S2. Pairwise genetic differentiation (*F_ST_*) among *Malus sieversii* sites (*N*>6)

|  | Kaz_Kuz | Kaz_3 | Kaz_djun | Kaz_Aksu | Ch_Xinj | Kaz_taut |
| --- | --- | --- | --- | --- | --- | --- |
| Kaz_3 | -0.005 |  |  |  |  |  |
| Kaz_djun | 0.0336*** | 0.0152*** |  |  |  |  |
| Kaz_Aksu | 0.0574*** | 0.0317*** | 0.0536*** |  |  |  |
| Ch_Xinj | 0.0343*** | 0.0142*** | 0.0262 | 0.0172 |  |  |
| Kaz_taut | 0.023*** | 0.0151* | 0.0063 | 0.0391*** | 0.0076** |  |
| Kaz_unid | 0.0183 | 0.0203 | 0.0017 | 0.06*** | 0.0157** | -0.008 |
| Kaz_Kokb | 0.0157*** | -0.0037 | 0.0102 | 0.0515*** | -0.0021 | -0.0016 |

*0.05<P≤0.01 ; ** 0.01<P≤0.001 ; *** P<0.001

Table S3: Pairwise genetic differentiation (*F_ST_*) among *Malus orientalis* sites (*N*>6)

| sites | ARA | Djermuk1 | Djermuk2 | Hermon1 | Hermon2 | Hermon3 | Jermouck | KhosrovR1 | KhosrovR5 | KhosrovR6 | Shikahog1 | Shikahog3 |
| --- | --- | --- | --- | --- | --- | --- | --- | --- | --- | --- | --- | --- |
| Djermuk1 | 0.0258*** |  |  |  |  |  |  |  |  |  |  |  |
| Djermuk2 | 0.0293*** | 0.0010 |  |  |  |  |  |  |  |  |  |  |
| Hermon1 | 0.0282*** | 0.0123** | -0.0135 |  |  |  |  |  |  |  |  |  |
| Hermon2 | 0.0152 | -0.0051 | -0.0063 | 0.0044 |  |  |  |  |  |  |  |  |
| Hermon3 | 0.0324*** | 0.0127*** | 0.0068 | 0.0101 | 0.0167 |  |  |  |  |  |  |  |
| Jermouck | 0.0131* | 0.0120** | 0.0109 | 0.0129** | 0.0043 | 0.0255*** |  |  |  |  |  |  |
| KhosrovR1 | 0.0081 | 0.0222*** | 0.0256*** | 0.0242*** | 0.0179** | 0.0335*** | -0.0024 |  |  |  |  |  |
| KhosrovR5 | 0.0148*** | 0.0114 | 0.0273 | 0.0184* | 0.0200 | 0.0389* | 0.0135 | 0.0103 |  |  |  |  |
| KhosrovR6 | 0.0192 | 0.0123** | 0.0229*** | 0.0176*** | 0.0103*** | 0.0231*** | 0.0124*** | 0.0122 | -0.0031 |  |  |  |
| Shikahog1 | 0.0403*** | 0.0434*** | 0.0559*** | 0.0463*** | 0.0460*** | 0.0646*** | 0.0241*** | 0.0210*** | 0.0269* | 0.0371** |  |  |
| Shikahog2 | 0.0658*** | 0.0248** | 0.0563*** | 0.0504*** | 0.0530** | 0.0692*** | 0.0172* | 0.0273*** | 0.0449* | 0.0471*** | 0.0078 |  |
| Vorotanp1 | 0.0126 | -0.0007 | -0.0005 | 0.0026 | -0.0155 | 0.0145* | 0.0045 | 0.0093 | 0.0030 | 0.0044 | 0.0363*** | 0.0371*** |
| Vorotanp2 | 0.0312*** | 0.0196*** | 0.0048* | 0.0083** | -0.0017 | 0.0225*** | 0.0260*** | 0.0311*** | 0.0251** | 0.0215** | 0.0594*** | 0.0574*** |

*0.05<P≤0.01 ; ** 0.01<P≤0.001 ; *** P<0.001

Figure S1. Bayesian clustering results for *Malus sieversii* in Central Asia (*N*=101) using the program TESS from *K*=2 to *K*=6. Each individual is represented by a vertical bar, partitioned into *K* segments representing the amount of ancestry of its genome in *K* clusters. Visualization was improved by sorting genotypes by sites.

Figure S2. Bayesian clustering results for *Malus orientalis* in the Caucasus (*N*=217) using the program TESS from *K*=2 to *K*=6. Each individual is represented by a vertical bar, partitioned into *K* segments representing the amount of ancestry of its genome in *K* clusters. Visualization was improved by sorting genotypes by sites.

Figure S3. Maps representing the mean membership proportions for *K* clusters, for samples of *Malus sylvestris* collected from the same site. Membership proportions were inferred with the Bayesian clustering algorithm implemented in TESS. At *K*=5 the results presented are those of the minor clustering solution (“mode”), showing the geographic location of the fourth previously identified cluster (Cornil*le et a*l., 2012).

**References**

Cornille A, Giraud T, Bellard C*, et al.* (2012) Post-glacial recolonization history of the European crabapple (*Malus sylvestris* Mill.), a wild contributor to the domesticated apple. *Molecular Ecology* **in press**.
